# Preparation and Characteristics of MAPbBr_3_ Perovskite Quantum Dots on NiO_x_ Film and Application for High Transparent Solar Cells

**DOI:** 10.3390/mi9050205

**Published:** 2018-04-27

**Authors:** Lung-Chien Chen, Kuan-Lin Lee, Chun-Yuan Huang, Jia-Ching Lin, Zong-Liang Tseng

**Affiliations:** 1 Department of Electro-Optical Engineering, National Taipei University of Technology, Taipei 10608, Taiwan; zwl44@yahoo.com.tw (K.-L.L.); t104658082@ntut.edu.tw (J.-C.L.); tw78787788@yahoo.com.tw (Z.-L.T.); 2 Department of Applied Science, National Taitung University, Taitung 95092, Taiwan; laputa@nttu.edu.tw

**Keywords:** MAPbBr_3_ perovskite, transparent solar cells, NiO_x_, quantum dots

## Abstract

In this work, a MAPbBr_3_ quantum dot (QD-MAPbBr_3_) layer was prepared by a simple and rapid method. Octylammonium bromide (OABr) gives the MAPbBr_3_ better exciton binding energy, good surface morphology, and stability. To form a nanocrystalline thin film on indium tin oxide (ITO) glass, the QD-MAPbBr_3_ film was coated by a spin-coating method in a nitrogen-filled glove box and the NiO_x_ film was used as an adhesive layer and hole transport layer. The highest transmittance of MAPbBr_3_ on NiO_x_/ITO glass was around 75% at 700 nm. This study also reported a high transparent and perovskite bulk-free ITO/NiO_x_/QD-MAPbBr_3_/C_60_/Ag solar cell where the NiO_x_, QD-MAPbBr_3_, and C_60_ were used as a hole transport layer, active layer, and electron transport layer, respectively.

## 1. Introduction

**Figure 1:**
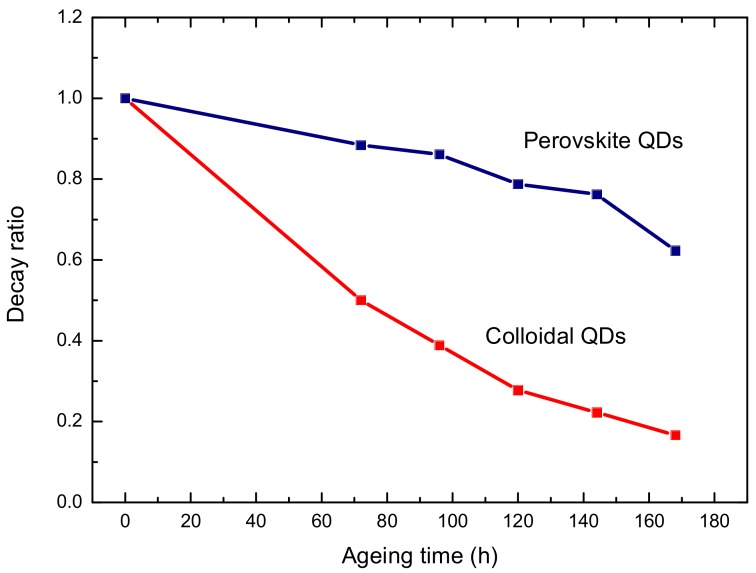
Ageing test of perovskite and colloidal of quantum dots (QDs) using photoluminescence.

The power conversion efficiency of perovskite solar cells has increased from 3.8% in 2009 to 22.1% in several years [1,2,3,4,5,6,7]; improvements in the efficiency of these solar cells have thus been remarkably rapid. Perovskite cells are also more cost effective than silicon cells and are easy to fabricate. Perovskites generally have an ABX_3_ structure (A = cationic organic molecule; X = halide) [8,9]. These materials have a number of unique attributes including bipolarity, high absorption coefficients, long carrier diffusion lengths, and high solar conversion efficiencies [10,11,12]. Furthermore, the sources of perovskite components are relatively abundant. A number of studies have been conducted on perovskite quantum dots. For example, Im et al. proposed a QD-MAPbI_3_ solar cell [13], and Huang et al., developed a QD-MAPbI_3_ light-emitting diode [14]. The perovskite material used in this experiment was QD-MAPbBr_3_. When compared to crystal MAPbBr_3_ films, QD-MAPbBr_3_ has unique physicochemical characteristics such as tunable band gaps, a functionalizable surface, and high quantum yields [15]. However, QD-MAPbBr_3_ has poor surface morphologies; these traits can be expected to affect the efficiencies of the solar element. Recent studies have shown that reductions in the sizes of perovskite crystals effectively reduce the number of internal defects in the perovskite material and the probability of non-radiative transitions. This was shown to improve the fluorescence efficiency of the QD-MAPbBr_3_ material as well as its stability [16,17]. Several articles about colloidal QD solar cells have been reported [18,19,20], however, in our work comprising the perovskite QDs (MAPbI_3_), the colloidal QDs (ZnSe) demonstrated poor reliability, as shown in Figure 1. Even so, QD-MAPbBr_3_ is still far away from commercial applications. In this work, octylammonium bromide (OABr) was added to QD-MAPBr_3_ to improve its exciton binding energies, surface morphology, and stability to improve the efficiency of QD-MAPBr_3_ devices.

## 2. Materials and Methods 

**Figure 2:**
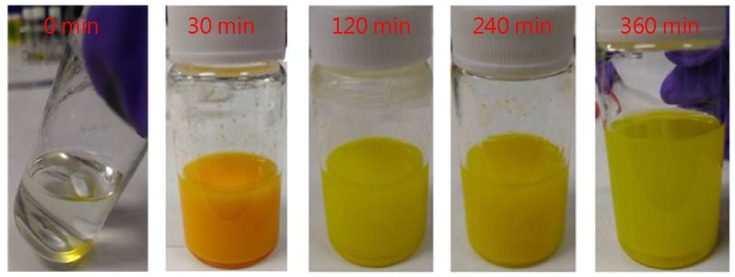
Pictures of QD-MAPbBr_3_ solutions for different reaction times.

First, a MAPbBr_3_ perovskite quantum dot solution was prepared. OABr powder (37.8 mg) was added to a solution containing 6 mL of octadecene and 60 µL of oleic acid. A methylammonium bromide (MAB) solution (13.2 mg of MAB dissolved in 300 µL of dimethylformamide (DMF) solvent) and PbBr_2_ solution (110.1 mg of PbBr_2_ dissolved in 500 µL of DMF solvent) were then added to this mixture to form the MAPbBr_3_ precursor solution (with 30, 120, 240, or 360 min of reaction time), as shown in Figure 2. Acetone was then added, followed by centrifugation to isolate the yellow precipitate from the MAPbBr_3_ precursor solution. The yellow precipitates were then dried under vacuum for half a day to completely remove the solvents of the precursor solution. The MAPbBr_3_ powder was then dissolved in hexane to prepare the MAPbBr_3_ perovskite quantum dot solution. The detail of the QD-MAPbBr_3_ process has been reported elsewhere [21].

The ITO glass substrate was sonicated for 10 min in acetone, and then in ethanol, and finally isopropanol. A nitrogen gun was used to dry the substrate, which was irradiated in UV-ozone for 10 min. A 0.45-µm syringe filter was used to drop NiO_x_ evenly on the ITO substrate, followed by spin coating at 4500 rpm for 90 s. The substrate was then heated at 120 °C on a hot plate for 10 min, and baked in an oven at 350 °C for 10 min. The NiO_x_ film here was as an adhesive layer and hole transport layer to coat the QD-MAPbBr_3_ layer. The resulting NiO_x_/ITO substrate was placed in a glove box filled with nitrogen, and a micropipette was used to evenly drop 50 µL of QD-MAPbBr_3_ on the NiO_x_/ITO substrate. The substrate was then coated in two stages (the rotational speeds and times of the first and second stages were 1000 rpm for 10 s and 3000 rpm for 10 s, respectively). After the coating process was completed, the substrate was placed on a hot plate for heating at 80 °C for five minutes. Finally, C_60_ and Ag were then thermally deposited one after the other in a thermal evaporator with a high-vacuum environment (1.5 × 10^−6^ torr) to complete the whole cell structure. In this device, NiO_x_ was the hole transport layer, QD-MAPbBr_3_ was the absorption layer, and C_60_ was the electron transport layer. The current density–voltage (J–V) curves of the photovoltaic cells were obtained using a source-measurement unit (2400, Keithly, Cleveland, OH, USA). The optical intensity of the simulated sunlight was calibrated using a reference cell (91150V, Oriel, Strarford, CT, USA) with an optical filter (KG-5) to have an intensity of 100 mW/cm^2^. The absorbance spectra were determined by a visible spectrometer. The crystallinities of MAPbBr_3_ thin films were determined by a commercial X-ray diffractometer (PW-1830, PANalytical, Almelo, The Netherlands). The photoluminescence (PL) spectra were measured with a commercial PL spectrometer (Protrustech Co., Ltd., Taipei, Taiwan).

## 3. Results and Discussion

**Figure 3:**
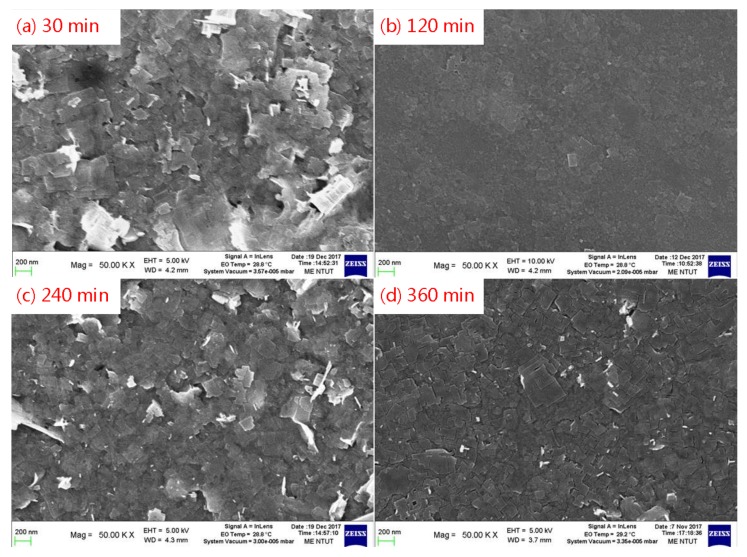
SEM images of the surface morphologies of the QD-MAPbBr_3_ films for the reaction times of (**a**) 30; (**b**) 120; (**c**) 240; and (**d**) 360 min.

**Figure 4:**
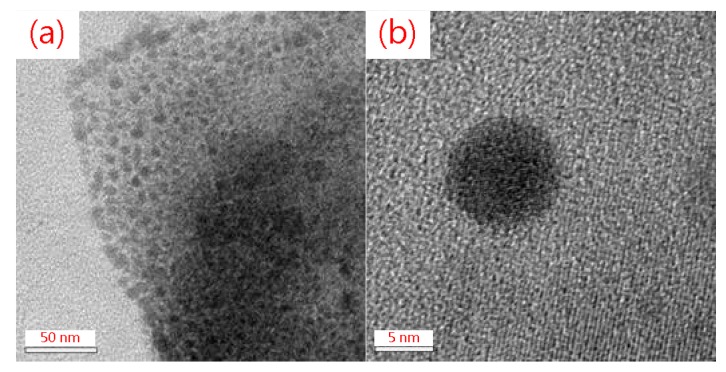
TEM images of the QD-MAPbBr_3_ (**a**) particle and (**b**) quantum dot.

Figure 3a shows a scanning electron microscopy (SEM, GeminiSEM, ZEISS, Oberkochen, Germany) image of the surface morphology of the QD-MAPbBr_3_ film with 30 min of reaction time. Here, it presented significant differences in the size between the grains of the film, and some of these grains were aggregated into QD-MAPbBr_3_ particles. This could be because the reaction time allowed for the reaction between PbBr_2_ and the mixture of MAB and medium-chain OABR in the presence of oleic acid and octadecene was insufficiently short in this case, thus the reactions at each grain were still incomplete. Figure 3b exhibits the smallest and most homogeneous grains among the four reaction times. The QD-MAPbBr_3_ particles that covered the NiO_x_ layer were smooth and uniform and formed a continue film. This indicated that 120 min of reaction time was the optimal reaction time. However, as the reaction time increased to 240 min, the OABr precipitated on the surface, causing some of the grains to aggregate into a sheet-like film, as shown in Figure 3c. When the reaction time was increased to 360 min, the surface morphology was a continued film due to the grain aggregation of most of the QDs by the formation of a homogeneous solution, as shown in Figure 3d. Figure 4a presents a transmission electron microscopy (TEM, Tecnai F30, Philips, Germany) image of the QD-MAPbBr_3_ particle consisting of many quantum dots. The sizes of the quantum dots ranged between 5 nm and 7 nm, as shown in Figure 4b. The QD-MAPbBr_3_ particle formed from many MAPbBr_3_ quantum dots due to aggregation. However, there was no reaction between the quantum dots.

**Figure 5:**
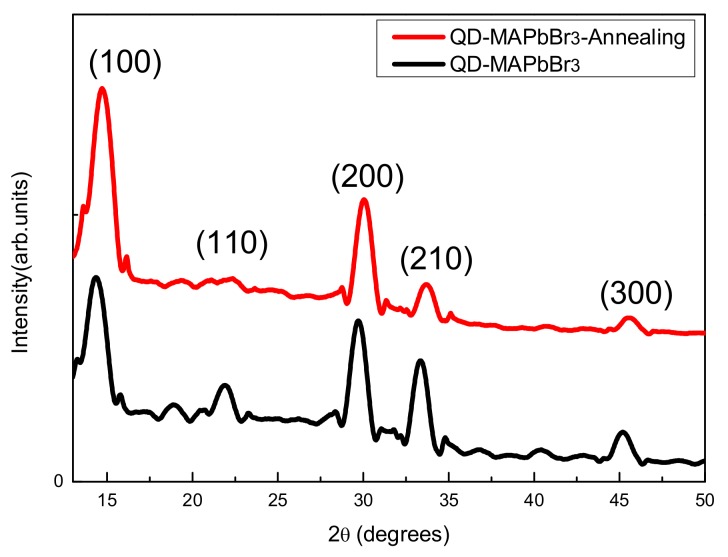
XRD patterns of the QD-MAPbBr_3_ with and without annealing treatment.

Figure 5 shows the XRD patterns of the QD-MAPbBr_3_ films. The QD-MAPbBr_3_ films were deposited on the NiO_x_ layer for all measurements in this study. The diffraction peaks occurred at 14.53°, 22.04°, 29.7°, 33.5°, and 45.35°, and these reflections corresponded to the (100), (110), (200), and (300) crystal lattice planes of the MAPbBr_3_, respectively [22,23]. After annealing, XRD peaks of the MAPbBr_3_ films were slightly shifted towards higher diffraction angles. Two diffraction peaks with the most intensities at 14.53° and 29.7° were significantly increased after the annealing process. This indicated that the annealed film had smaller crystal lattice distances and greater crystallinity than that of the film without annealing treatment.

**Figure 6:**
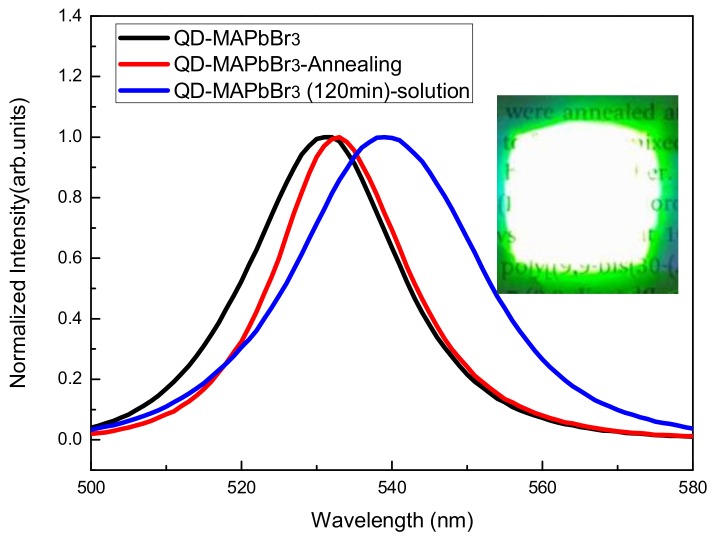
PL spectra of the QD-MAPbBr_3_ films with and without annealing treatment. The inset shows a picture of the QD-MAPbBr_3_ film excited by a 405-nm laser.

**Figure 7:**
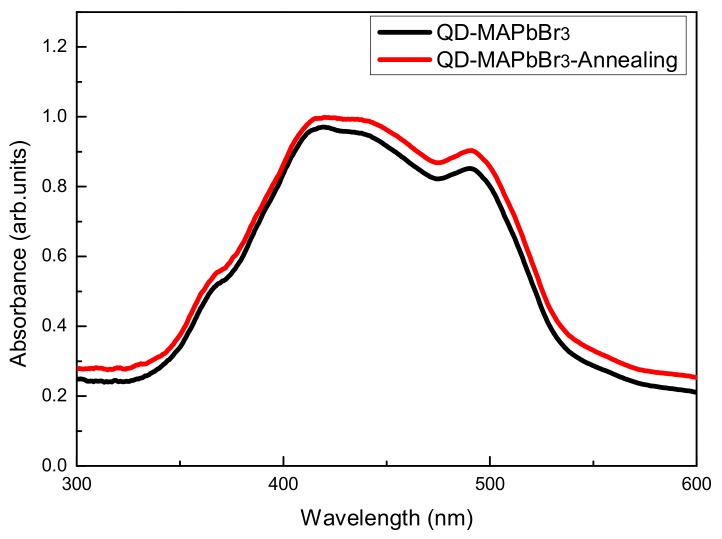
Absorption spectra of the QD-MAPbBr_3_ films with and without annealing treatment.

**Figure 8:**
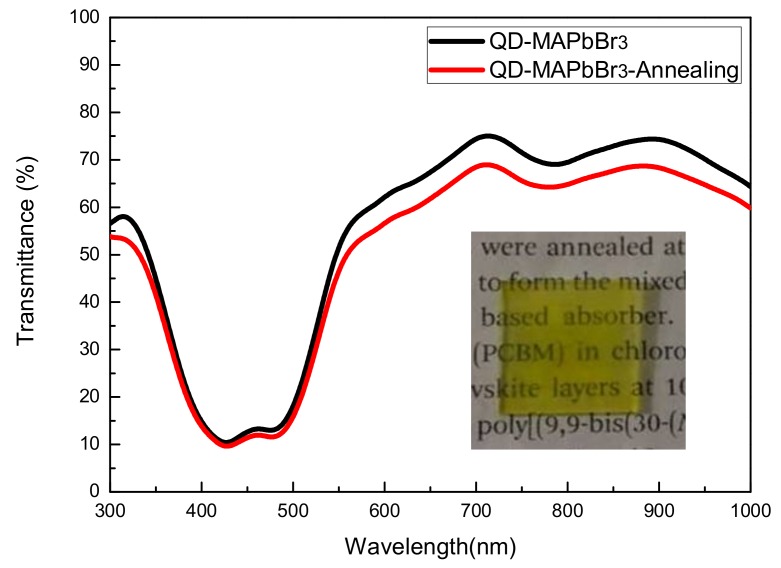
Transmittance spectra of the QD-MAPbBr_3_ films with and without annealing treatment. The inset shows the picture of the QD-MAPbBr_3_ film.

Figure 6 shows the photoluminescence (PL) spectra of QD-MAPbBr_3_; the locations of peaks of the QD-MAPbBr_3_ solution, the film without annealing, and the annealed film were at 539, 531, and 533 nm, respectively. The band gap can be expected to increase when the size of the quantum dots becomes smaller, thus leading to a blue shift of the PL emission peak. In this work, the PL spectrum of the film without annealing exhibited a greater blue shift than that of the film with annealing. This indicated that the film without annealing had a smaller particle size and larger band gap than the film with annealing. The inset is a picture of a QD-MAPbBr_3_ specimen that emitted green light while being excited by a 405-nm laser. The fluorescence quantum yield is around 4.96%. It reveals a high trap density in the QD-MAPbBr layer [24,25,26,27]. Figure 7 shows the absorption spectra of the QD-MAPbBr_3_ films. One may observe that the films absorbed significantly between 400 nm and 500 nm, and the absorption increased slightly after film annealing. When compared to conventional MAPbBr_3_ films, the absorption peak of the QD-MAPbBr_3_ films underwent a blue shift to 491 nm due to the quantum confinement effects in MAPbBr_3_ [28]. Figure 8 shows the transmittance spectra of QD-MAPbBr_3_ with and without annealing. The transmittance of the film was 65% between 600 nm and 1000 nm, but only 10% between 400 nm and 500 nm. The highest transmittance was around 75% at 700 nm in the spectrum of QD-MAPbBr_3_ on NiO_x_/ITO glass. The location of the absorption edge was at 500 nm, corresponding to the absorption of the QD-MAPbBr_3_ films. The inset in Figure 8 is an image of the QD-MAPbBr_3_ film and shows that the text below the film was easily readable. 

**Figure 9:**
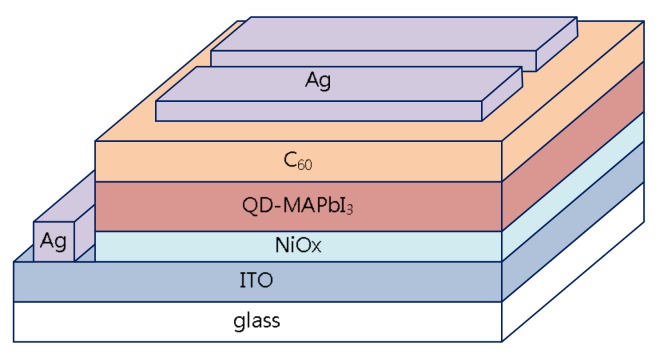
Structural diagram of the QD-MAPbBr_3_ solar cell.

**Figure 10:**
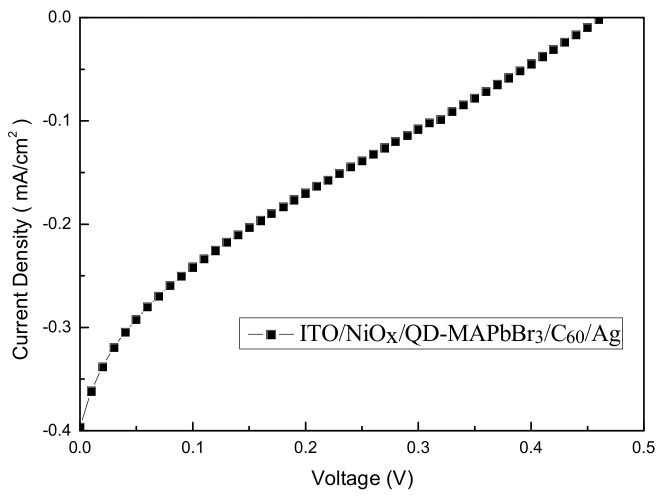
Typical J–V curve of the ITO/NiO/QD-MAPbBr_3_/C_60_/Ag structure solar cell.

Figure 9 illustrates the structure of a QD-MAPbBr_3_ solar cell device. Figure 10 presents a typical J–V curve of the ITO/NiO/QD-MAPbBr_3_/C_60_/Ag structure solar cell. The open circuit voltage (V_oc_), current density (J_sc_), fill factor (FF), and power conversion efficiency (PCE, %) of the solar cell were 0.04 V, 0.257 mA/cm^2^, 0.258, and 0.0347, respectively. The QD-MAPbBr_3_ solar cell exhibited poor performance due to the high resistance of the QD-MAPbBr_3_ film. This may have contributed to the electron transition in the QD-MAPbBr_3_ film being hard from quantum dot to quantum dot, from grain to grain, and from particle to particle, as shown in Figure 3 and Figure 4.

## 4. Conclusions

This work involved varying the reaction time of the reaction between MAB, PbBr_2_, and OABr to optimize the size of the precipitating particles of the resulting QD-MAPbBr_3_ solution; this solution was then spin-coated to form a film and was used as the active layer of a perovskite solar cell. The transmittance spectra showed that the QD-MAPbBr_3_ films had high levels of transmissivity, and their PL spectra also indicated a significant degree of fluorescence. The TEM image showed that these films consisted of nanocrystals with a size of approximately 6 nm. However, the J–V curve and PCE values of the film showed that the FF of the resulting solar cell was poor. The QD-MAPbBr_3_ solar cell exhibited a high transparency, but poor performance. Electron transition in the QD-MAPbBr_3_ film was hard from quantum dot to quantum dot, from grain to grain, and from particle to particle. Therefore, the QD-MAPbBr_3_ film requires further refinement to improve its quality, as the maximum solar conversion efficiency of the film is only 0.0347% at present.
